# Factors associated with the length of stay in emergency departments in Southern-Ethiopia

**DOI:** 10.1186/s13104-019-4271-7

**Published:** 2019-04-25

**Authors:** Getahun H/meskel Alemu, Keneni Gutema Negari, Kaleb Mayisso Rodamo, Agete Tadewos Hirigo

**Affiliations:** 1 College of Medicine and Health Science, Comprehensive Specialized Hospital, Hawassa University, P.O. Box 1560, Hawassa, Ethiopia; 2 College of Medicine and Health Science, School of Public Health, Hawassa University, Hawassa, Ethiopia; 3 College of Medicine and Health Science, School of Medical Laboratory Sciences, Hawassa University, Hawassa, Ethiopia

**Keywords:** Emergency department, Length of stay, Hawassa, South-Ethiopia

## Abstract

**Objectives:**

This cross-sectional study was conducted on 399 patients at Hawassa University Comprehensive Specialized Hospital from February 15 to March 30/2018 to assess the length of stay (LOS) and its associated factors in emergency departments (EDs).

**Result:**

About 91.5% patients were stayed in the EDs for greater than 24 h in different reasons. Inadequacy of beds in inpatient wards, overcrowding, absence of different laboratory test profiles and delay in radiological services were showed a significant differences in LOS greater than 24 h when compared to LOS ≤ 24 h in EDs (p < 0.05 for all). In addition, admission beds [adjusted odds ratio: 8.7 (95% CI 3.2–23.2)]; overcrowding [adjusted odds ratio: 3.6 (95% CI 1.6–8.3)]; laboratory test profiles [adjusted odds ratio: 5.1 (95% CI 1.9–14.1)], and radiology services [adjusted odds ratio: 3.7 (95% CI 1.5–9.2)] were significantly and positively associated with LOS greater than 24 h in EDs. Further, a significant proportion of patients were stayed for unnecessary extended length of time in EDs due to different factors. Therefore, the commitment of organization is crucial to provide sufficient number of admission beds, to scale-up laboratory test profiles and to decrease radiology service turn-around time in order to improve LOS in EDs.

## Introduction

Length of stay (LOS) in the hospital is a vital indicator of health services that is used to evaluate the efficacy, patients care, organizational management and health care system. In addition, shorter hospital stays reduce unnecessary medical fees and upsurge the bed turnover rate that helps to rise the profit margin of institution, in addition to dropping of total social related costs [1–3].

The emergency departments (EDs) care demand is affected by different factors that include social, epidemiological problems and features related to the organization of the health system and inadequate services organizing [4, 5]. ED overcrowding is a global worry and signifies worldwide crisis that may affect access to health care and quality of service delivery for patients [6–8]. In ED working area, staffs may face different challenges such as unanticipated states of patients’ illness, unclear patient medical histories, and the necessity for time-dependent and triage-based judgment creation [9]. Further possible mechanisms for the prolonged LOS may include lack of decision-making, reluctance to order tests or consultations that may take a long time, unfinished investigation, inadequate monitoring, incomplete treatment or a lack of plan to discharge and follow-up preparations [10, 11].

Data regarding LOS in hospital EDs in Ethiopia is scarce, as well the nature and burden of prolonged LOS has not been well studied in emergency cases. Therefore, this study aimed to assess the pattern and associated factors of patients LOS in the ED.

## Main text

### Study settings and study population

This cross-sectional study was conducted at Hawassa University comprehensive specialized hospital in Hawassa town, Southern Ethiopia. The Hospital was established in November 2005 for the purpose of health professionals training and health care service delivery. It has about 400 beds; and expected to serve more than 18 million of people in Southern-Ethiopia as well as neighboring regions. Currently there are 525 administrative staffs and 793 health professional staffs working in the hospital. In addition, around 29 and 48 nurses are working in pediatrics and adult emergency departments, respectively. In total, there are 62 beds in both EDs (30 in pediatrics and 32 in adults). Moreover, the study was conducted from February 15 to March 30/2018 on 422 patients in EDs to assess LOS and its associated factors. Due to lack of similar studies in Ethiopia, the sample size was determined by considering 50% proportion using single population proportion formula at 95% confidence interval (CI).

$${\text{n}} = \, \frac{{\left( {{\text{Z}}_{\upalpha/2}} \right) \times {\text{p}}\left( {\text{q}} \right)}}{{{\text{d}}^{2} }} = \frac{(1.96)^2 \times 0.5(1-0.5)}{(0.05)^2} = 384$$

where n is the required sample size, Z_α/2_ (Z Alpha over Two) = 1.96 at 95% CI, p is the proportion of LOS, q = 1 − p and d is the assumed marginal error (5%). In addition, including with 10% non-response rate [384 + (384 × 0.1)], the final sample size calculated to be 422. To allocate the number of participants from pediatrics and adult EDs, a one-year emergency attendants’ records was assessed and the trend showed 9200 cases (912 pediatrics and 8288 adults) were visited in year 2017. Based on this, sample size distribution was done as follows: 42 cases [(912/9200) × 422] from pediatrics ED and 380 cases [(8288/9200) × 422] from adults ED were expected to be included in the study. Finally, patients were selected using systematic random sampling technique from these departments for the study. Moreover, socio-demographic and others relevant clinical information of the study subjects were collected using pre-tested and an interviewer directed structured questionnaires and the interview was done by trained nurses who were working in the EDs. Furthermore, the interview was conducted directly to patients for those who were conscious and able to communicate properly while patient’s relatives/families were interviewed for those patients who were unconscious and unable to communicate. Data collection process was conducted for 24 h during data collection period in three shifts. All patients visited adult and pediatric EDs during the study period were eligible for the study. While patients visited the EDS and referred to other health facilities before completing their medical care and patients directly sent to inpatient wards for admission were excluded from the study.

#### Definition and assessment criteria of LOS

LOS is the total length of time for all emergency case attendants who stay in the emergency room from minutes to hours (days) starting from login. According to Ethiopian hospital services transformation guidelines: patients’ LOS in the emergency department should *not be greater than* 24 h. Then transfer to wards has been facilitated before 24 h for proper inpatient admission if necessary [12].

### Statistical analysis

Entirely questionnaires were visually checked, coded and entered into epidata version 3.1 and then exported to statistical Package for Social Sciences (SPSS) version 20 for analysis. To explain study population in relation to relevant variables, descriptive statistics (frequencies, means and percentage) were used. In addition, significances in between two categorical variables were assessed by Chi-square test. Univariate and multivariate logistic regression was used to evaluate the association between LOS and independent variables. Moreover, a p-value < 0.2 was a cut-off to include variables for multivariate analysis and; in all situations results were accepted as statistically significant when a p-value is < 0.05 at 95% CI.

### Results

Totally 422 emergency cases were approached, of them 23 (5.5%) refused to take part in the study. About 57.6% (230/399) were males and the rest were females. The mean (± SD) age of the study participants’ was 38 (± 1.6) years with the range of 10–90 years (Table 1).

**Table 1:** Socio-demographic characteristics of the study population

Variable	No. (%)
Gender	
Female	169 (42.4)
Male	230 (57.6)
Age in years, (mean ± SD), range (years)	38.1 (1.6), (10–96)
≤ 10	1 (0.3)
11–20	53 (13.3)
21–30	118 (29.6)
31–40	76 (19)
41–50	76 (19)
> 50	75 (18.8)
Educational level	
Formal education	154 (38.6)
Non formal education	245 (61.4)
Ethnicity	
Oromo	128 (32.1)
Amhara	34 (8.5)
Wolayita	42 (10.5)
Sidama	133 (33.3)
Others	62 (15.5)
Residence	
Urban	113 (28.3)
Sub-urban	130 (32.6)
Rural	156 (39.1)
Region	
Oromia	177 (44.3)
South-Ethiopia	215 (53.7)
Others	7 (1.8)

Patients LOS in the EDs for 24 h–4 days, 5–7 days, 8–10 days and more than10 days was 37.2%, 42.6%, 7.8% and 4.0%, respectively. In addition, the trend indicated that 91.5% of the subjects were stayed in EDs for more than 24 h (days to weeks). Majority of the patients (73.7%) were examined by interns while 10.8% and 0.8% were examined by residents and specialists, respectively. Regarding investigations, 49.1% (196) were sent to Lab and 50.9% (201) were sent to radiology rooms for X-ray, MRI, Ultrasound and CT scan diagnosis. In addition, 57.1% (232) investigations were done in the hospital while the rest 41.1% (167) investigations were performed in private clinics and private hospitals. About 60 patients (15%) have accessed their diagnostic results within 1 h while 98 (24.5%) have accessed after 24 h. About 38.1% (152) of the patients were not satisfied by the hospital services due to different reasons. Furthermore, the shortage of beds in inpatient wards, overcrowding, shortage of lab test menu and delay in radiological services were showed a significant differences with LOS greater than 24 h when compared to LOS ≤ 24 h in EDs (Table 2).

**Table 2:** Distribution of factors related with length of stay in emergency department

Variable	≤ 24 h 34 (%)	> 24 h 365 (%)
Delay decision		
Yes	18 (52.9)	167 (45.8)
No	16 (47.1)	198 (54.2)
Shortage of inpatient beds		
Yes	7 (20.6)	21 (58.6)
No	27 (79.4)	151 (41.4)**
Overcrowding		
Yes	15 (44.1)	256 (70.1)
No	19 (55.9)	109 (29.9)*
Cancelation and delay of surgery		
Yes	15 (44.1)	176 (48.2)
No	19 (55.9)	189 (51.8)
Delay of laboratory diagnosis		
Yes	12 (35.3)	187 (51.2)
No	22 (64.7)	175 (48)
Delay of consultation		
Yes	23 (69.7)	231 (63.3)
No	10 (30.3)	134 (36.7)
Shortage of laboratory test menu		
Yes	7 (20.6)	176 (48.2)
No	27 (79.4)	189 (51.8)*
Delay in radiological service		
Yes	9 (26.5)	207 (56.7)
No	25 (73.5)	158 (43.3)**

* p < 0.01, ** p < 0.001

Multivariate logistic regression was used to evaluate the association of independent variables with LOS in the EDs. Overcrowding, shortage of lab test menu, delay of lab diagnosis, delay in radiological services and shortage of beds were significantly associated with extended LOS in the EDs (Table 3).

**Table 3:** Factors associated with length of stay in emergency department

Variable	Length of stay in emergency at 95% CI	
	Response	AOR (95% CI)
Decision delay	Yes	0.33 (0.1–0.9)*
	No	1.00
Overcrowding	Yes	3.6 (1.6–8.3)**
	No	1.00
Cancelation and delay of surgery	Yes	1.425 (0.62–3.2)
	No	1.00
Delay of lab diagnosis	Yes	4.5 (1.4–13.9)**
	No	1.00
Delay of consultation	Yes	0.447 (0.10–1.1)
	No	1.00
Shortage of laboratory test menu	Yes	5.1 (1.9–14.1)**
	No	1.00
Delay in radiological services	Yes	3.7 (1.5–9.2)**
	No	1.00
Shortage of beds in inpatient wards	Yes	8.7 (3.2–23.2)***
	No	1.00

* p < 0.05, ** p < 0.01, *** p < 0.001

## Discussion

The prevalence of LOS greater than 24 h in emergency rooms was 91.5%. This was occurred due to different reasons, and some of the reasons were shortage of beds in inpatient wards, overcrowding, absence of different lab test profiles and delay in radiological services and it accounts 58.6%, 70.1%, 48.2%, and 56.7%, respectively.

In addition, these depicted variables were significantly higher (p < 0.05) in emergency patients with prolonged LOS in EDs. Moreover, overcrowding, inadequacy of lab test profiles, delay in lab diagnosis, occupancy of inpatient beds, and delay in radiological services were significantly and positively associated with prolonged LOS in the EDs.

Different countries have different rate of LOS in the EDs; they may have shorter LOS and separate management system based on the status of their facility when compared to our settings. In addition, proportion of LOS in ED is vary from one country to another according to their cutoffs and several studies highlighted different proportions, like 8.2% in Taiwan [13], 10.2% in Iran [14] and 42.1% in Brazil [15]. However, the present study showed that 91.5% of prolonged LOS in ED according Ethiopian hospital services Transformation guidelines criteria. The variation could be attributed to differences in organization structure, administration, the health system of organization and the availability of organized services.

We found that significantly higher prevalence of prolonged LOS in delayed radiological services and shortage of lab test profiles. In addition, these depicted variables were significantly associated with extended LOS in emergency rooms. The finding was in line with the study reported by Yoon et al. [16]. The ED overcrowding has different factors and which is initiated by accident conditions, fighting, consequential lodging of admitted patients in the wards and low rate of patients discharge in the wards [17]. Our study indicated that the rate of overcrowding was high and it associated with prolonged LOS in the EDs. In support, the report from Rwanda indicated that 63% of respondents perceived increasing complexity and perception of cases as a cause of overcrowding [18]. Besides, different studies reported that overcrowding is a global challenge and may causes crisis in the provision of health care and quality service delivery [6–8, 19, 20]. Further, the consequence of ED crowding is associated with the probability of poor performance and adverse clinical outcomes including mortality [21].

The present study showed that lack of admission beds in inpatient wards was the one factor that extends LOS and it is associated with LOS > 24 h. In provision, the study reported by Richardson indicated that one of the direct influencing factors of LOS in emergency department was identified as “access block, [22], that refers to the situation in which patients requiring an emergency hospital admission endure more than 8 h in the emergency department due to a lack of access to appropriate hospital inpatient beds [22, 23].

In conclusion, the findings revealed that the length of stay in emergency rooms was high. Overcrowding, delay in lab diagnosis, inadequacy of laboratory test profiles, delay in radiological services and inadequacy/occupancy of admission beds in inpatient wards were found to have a significant association with prolonged LOS in the EDs. In addition, this indicates majority of emergency patients might face different challenges, like psychological, socio-economic and general health care problems. This prolonged LOS alarms the responsibility of administrative bodies of the organization and other stakeholders to maintain necessary infrastructures. Moreover, the commitment of health care providers’ is also critical to shorten patients LOS in the EDs. Furthermore, we recommend further deep study to address whether an underlying association exists between concealed variables and prolonged LOS in ED using qualitative approaches, advanced and data based interventions in order to improve LOS in EDs.

## Limitations

First, our study was a cross-sectional by nature and it cannot provide sufficient evidence of causality concerning LOS in EDs and its associated factors. Second, we analyzed LOS of EDS visit in a restricted period and we concentrated on a solitary EDs in Hawassa University Comprehensive Specialized Hospital that probably functions vary from other EDs in Hawassa city health institutions and somewhere else in the country. Third, we did not assess the communication gaps in between patients or patient relatives and health caregivers to address whether LOS can be influenced by the communication barrier or not. Irrespective of the portrayed limits, this study ultimately enhances helpful information on LOS in a scarce data condition of Africa, particularly in Ethiopia including the study area.

## Abbreviations

ED: emergency department
LOS: length of stay
SPSS: statistical package for social sciences
